# Patronus: patient-specific training setup for aortic aneurysm repair

**DOI:** 10.1007/s11548-026-03715-y

**Published:** 2026-06-03

**Authors:** Henry John Krumb, Bernhard Dorweiler, Anirban Mukhopadhyay

**Affiliations:** 1 Computer Science Department, TU Darmstadt, Fraunhoferstr. 5, Darmstadt, 64293 Hessen Germany; 2 Klinik und Poliklinik für Gefäßchirurgie– Vaskuläre und endovaskuläre Chirurgie, Uniklinik Köln, Kerpener Str. 62, Cologne, 50937 Nordrhein-Westfalen Germany

**Keywords:** Endovascular Aneurysm Repair, Surgical Simulation, Patient-specific Treatment

## Abstract

**Purpose:**

Surgical training for Aortic Repair (EVAR) on simulators holds the potential to reduce radiation of the X-ray-guided procedure. Precision and realism of training simulators increases significantly with the use of patient-specific phantoms. However, radiation-free setups utilizing 3D-printed anatomies lack realistic imaging.

**Method:**

We propose PATRONUS, a hardware and software prototype for patient-specific, radiation-free surgical training for EVAR. 3D printed, transparent phantoms are imaged inside a black box which is accessed from the outside. Pseudo X-rays are generated through reconstructed radiographs, which are enhanced by virtual catheters and contrast agent.

**Results:**

Our prototype demonstrates a complete pipeline including a 3D printing workflow, phantom preparation, online registration of phantoms to the patient CT and pseudo X-ray generation. We report an average registration error (RMSE) of the scene inside the simulator to the digital twin of ${9.30}\,\hbox {mm}$. The entire simulator pipeline takes ${1.059}\,\hbox {s}$ to execute per image.

**Conclusion:**

State-of-the-art techniques for scene understanding and 3D printing enable the transition from simulated interventions on 3D printed vascular phantoms under cameras to a digital twin representation in the CT regime. The PATRONUS simulator prototype thus provides a foundation for radiation-free, realistic endovascular surgical training with patient-specific phantoms.

## Introduction

As endovascular procedures such as Aortic Repair (EVAR) are conducted under fluoroscopic X-ray imaging, vascular surgeons are exposed to high accumulated doses of ionizing radiation throughout their career [1]. Even though the *As Low As Reasonably Achievable (ALARA)* principle is established to minimize radiation and protective equipment is mandatory, the long-term radiation exposure still poses a significant risk to the health of surgeons [2].

Surgical training with simulators helps minimizing the overall duration of the intervention, and along with that the total fluoroscopy time [3], by preparing the surgeon for dexterous maneuvers and unforeseen problems. It was shown that simulation of EVAR can further increase the confidence of young surgeons practicing the procedure [4], which also reduces fluoroscopy time in the real intervention. While education about the ALARA-principle and safety measures already have a significant impact on the radiation exposure during a procedure [1] – a combination with *patient-specific and radiation-free* interventional training promises to further reduce radiation exposure.

Commercial radiation-free simulators for EVAR are often limited to a few pre-defined, artificial anatomies. *Patient-specific* EVAR training, while not to be found in commercial simulators, is already investigated in research settings. In earlier days, parameters of commercial simulators were tuned to those of real patients to increase the realism of the simulation [5]. More recently, thanks to recent technological advances, researchers started leveraging the potential of 3D printed patient-specific anatomies to increase realism. Torres and De Luccia conducted a study with rigid 3D printed anatomies under X-ray, showing that such a simulator setup was feasible [3]. Newer simulators are enhanced by perfusion systems to simulate blood flow under X-ray [6, 7].

However, 3D printed anatomies have a significantly different appearance under X-ray or cameras compared to the real patient vasculature under fluoroscopy. Most importantly, printed phantoms always have visible boundaries that do not appear in a realistic setting, which normally demands the injection of contrast agent to see the vascular anatomy and the spatial relation of tools within. Achieving realistic and *radiation-free* training in simulated EVAR with 3D printed anatomies is a challenge that has not been addressed in the literature yet. Combining 3D printing with computer vision and tool tracking is mainly challenging due to the complexity of the image processing and visualization pipeline. Further, a complete hardware setup has not yet been proposed in literature that is suited for the purpose of recording and tracking 3D printed phantoms. Since such processing is more intense than just displaying a fluoroscopic or webcam image, it is essential to develop a pipeline that works at high speed, preferably in real-time.

In this technical report, we present *PATRONUS*, a simulator setup prototype for EVAR that aims to bridge the gap between precise patient specific simulation and radiation-free training. Our simulator leverages state-of-the-art techniques for tracking, 3D reconstruction, registration and X-ray generation to build a self-contained black box simulator for endovascular surgery. 3D printed patient-specific phantoms are placed inside the black box simulator, which provides controlled lighting and has stereo cameras inside, from which the scene is reconstructed in order to estimate the phantom pose with respect to the camera. This pose is translated into the CT coordinate space in order to generate a realistic pseudo X-ray that the trainee uses for navigation.

The contributions of this work are three-fold:

1. We propose PATRONUS, a **pa**tient-specific **tr**aining setup f**o**r abdominal aortic a**n**e**u**ry**s**m repair. The setup is based on a simulator box equipped with cameras and a patient-specific 3D printed phantom inside.
2. On the software side, we propose a complete simulator pipeline that matches camera images with a digital twin to produce pseudo X-ray images. Pseudo X-rays of the scene inside the box are generated through 3D-3D registration followed by Digitally Reconstructed Radiograph (DRR) generation utilizing the estimated geometry. These pseudo X-rays are overlaid with the inserted materials. The pipeline is evaluated with respect to registration accuracy and processing time.
3. We publish a list of materials, downloadable 3D models of constructed parts and source code on the PATRONUS project page: https://patronus-simulator.de, in an effort to create a reproducible setup.

**Fig. 1:**
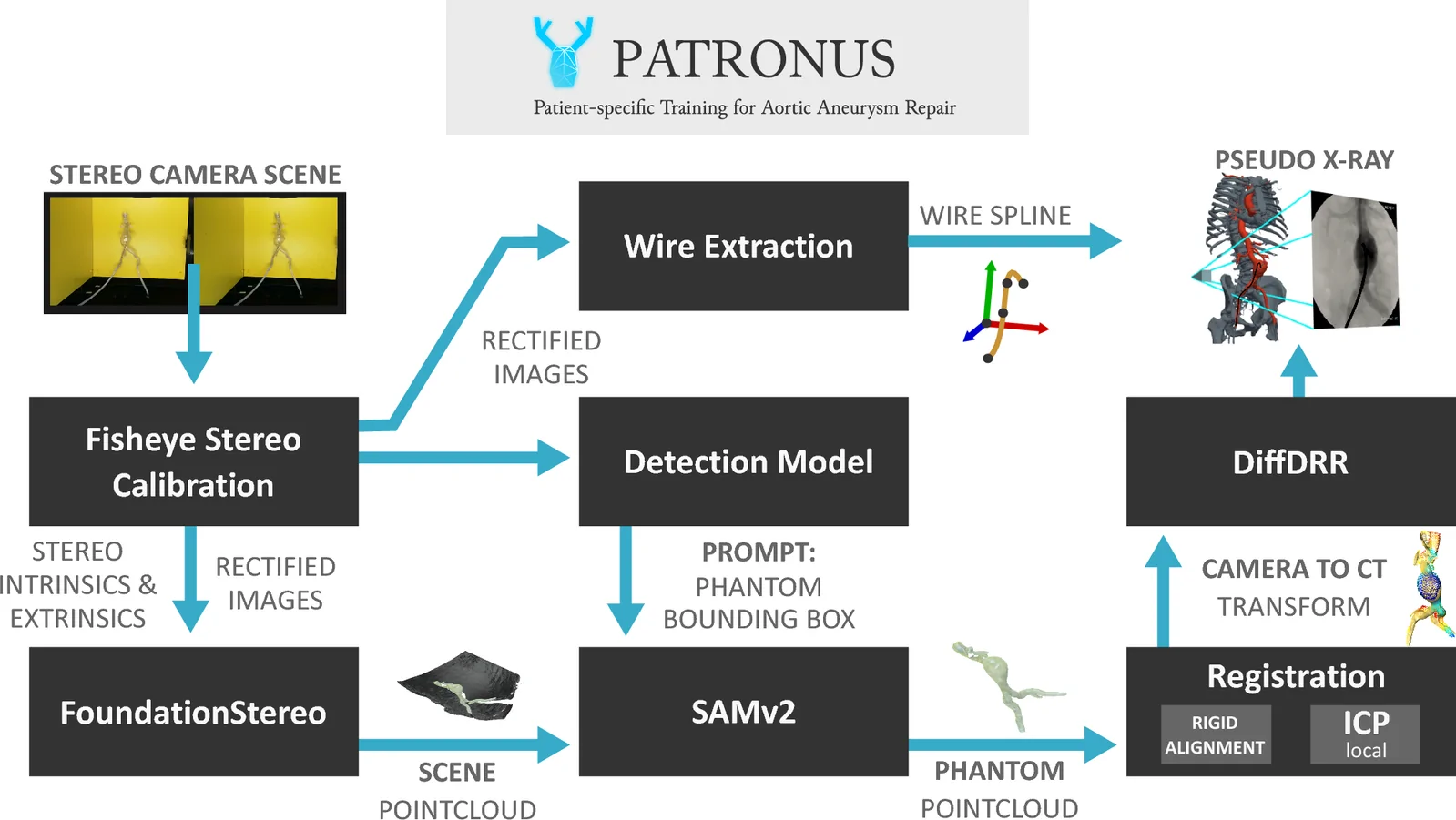
PATRONUS Pseudo X-ray generation pipeline for generating pseudo X-rays from the stereo camera scene. After calibration, stereo images are passed to 1. the wire extraction stage, and 2. stages for reconstructing the phantom as a 3D pointcloud (Yolo - SAMv2 - FoundationStereo)

## Related work

**Patient-specific EVAR Simulation:** As commercial interventional training simulators often lack flexibility and in most cases require a pre-defined set of anatomies, 3D printed vessel phantoms are becoming increasingly popular for patient-specific simulation in research settings. In 2019, the *Hamburg ANatomical NEurointerventional Simulator (HANNES)* was proposed as a patient-specific neurointerventional simulator that was recently enhanced by an EVAR module [7]. This module consists of a rigid 3D printed aortic phantom inside of a rigid fixture, that can be perfused with contrast agent for visibility under X-ray. A flexible, perfused model for thoracic EVAR was proposed by Mohl et al. [6], which has similar properties to human tissue under fluoroscopic X-ray, Ultrasound and CT angiography.

Until now, the use of 3D printed phantoms in a realistic interventional setting requires the use of radiation during training. Further, surrounding structures such as bones or metallic implants are not considered if only the aortic anatomy is imaged in an isolated fashion. However, navigating catheters and placing stent grafts inside a real vessel is more challenging, as the contours of the anatomy are only visible with contrast agent which is supposed to be injected sparsely. In this work, we propose the first setup which embeds 3D printed anatomies in a setting that mimics these constraints of a real intervention.

**Depth estimation for 3D scene understanding:** Until recently, accurate depth estimation from camera images used to be a major challenge in computer vision which was especially the case in scenes with transparent objects (like our aortic phantoms). Even with stereo cameras, generating a precise disparity map used to be difficult and resulting depth maps were often distorted and were hardly usable.

In the past few years, increasingly many zero-shot models for *monocular* depth estimation were proposed, such as DepthAnything v1 and v2 [8, 9], MiDas [10] or Geowizard [11]. In 2025, FoundationStereo was proposed [12] as a model that leverages stereo camera information and depth estimates from Depth Anything v2 to produce accurate disparity maps – even in scenes containing reflecting or transparent objects. PATRONUS makes use of these recent advancements in order to create a 3D reconstruction of the filmed phantom which is then mapped to the original CT space.

## Method

**Fig. 2:**
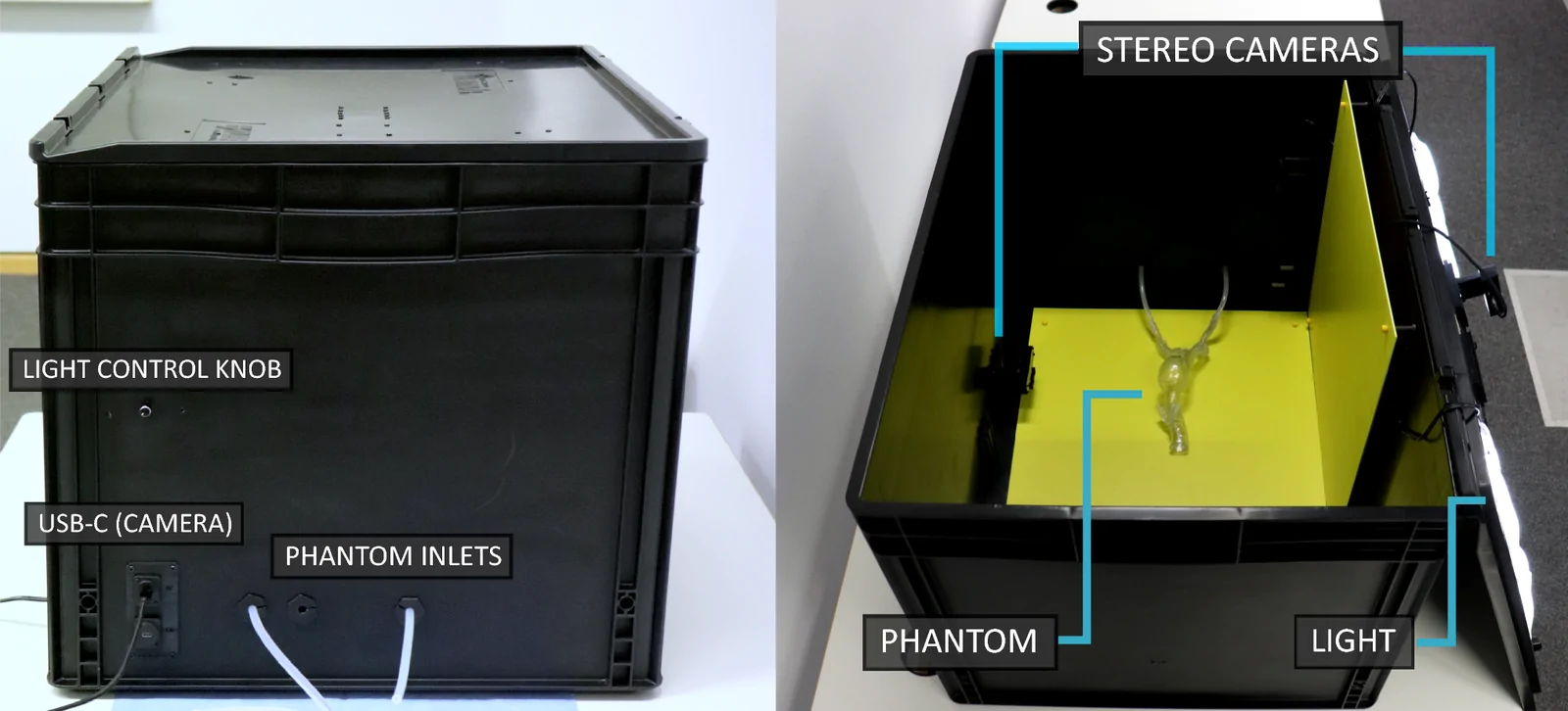
Black-box simulator setup. Left: Webcams inside are accessible through USB-C ports on the front panel. Grommets on the front panel can be used for phantoms of varying inter-iliac distances. Right: Phantom is placed inside the box

The simulator hardware setup consists of a black container with a stereo camera inside that images the phantom, catheters and guidewires during the training intervention (Fig. 2), which we further describe in Sec. 3.1. Since the patient-specific phantoms (described in Sec. 3.2) vary in size and anatomy, a complex computer vision pipeline is needed to track the phantom and materials inside and to reconstruct a digital twin of the scene that can be mapped to the space of the original CT (Fig. 1, Sec. 3.3). With this mapping, it is further possible to generate realistic pseudo X-ray images of the 3D printed patient (Sec. 3.4). Our software pipeline is implemented in Python, utilizing modules of the numpy [13, 14] and PyTorch [15] ecosystems, and Open3D [16].

### Black-box simulator

The hardware setup of the PATRONUS simulation environment comprises a black plastic box with a 1600×1200 pixel stereo camera (ELP 3D, 120 FPS global shutter, MJPEG coding) mounted on the inside, in which the 3D printed phantoms are placed, as shown in Fig. 2. The USB-C interfaces for the stereo camera is exposed to the outside, as well as flexible silicone tubes that are firmly connected to the iliac arteries of the phantom.

The stereo camera is attached to the top of the phantom, resembling AP (anterior-posterior) projection of an angiography suite or mobile C-arm. LED strips inside the simulator box, attached to the ceiling, provide controlled constant lighting. The cameras are equipped with a polarizing filter to limit the effect of specular reflections created by the ceiling light on the phantom surface.

The stereo camera setup is calibrated using an 8×5 checker board and a fisheye stereo camera model, which yields a good approximation of the camera intrinsics and extrinsics. In total, we collect 66 calibration frames with the board located at different positions, and calibrate the cameras with an error of ${0.267}\,\hbox {mm}$ total RMSE. Given that calibration, all images collected for training and evaluating the remaining steps of the simulator pipeline are undistorted and rectified.

To capture which patient’s anatomy is inserted into the simulator, an NFC sticker tag on the bottom of each phantom stores the respective patient identifier. An NFC reader attached to the phantom fixture is repeatedly queried by an ESP32 (Espressif Systems) via Serial Peripheral Interface (SPI), which continually transmits the patient ID to the host computer over a serial link. To mount the phantoms in the simulator box, each of them is equipped with two flexible silicone tubes connected to the iliac arteries with shrink-on PVC tubes.

### Patient-specific phantoms

**Fig. 3:**
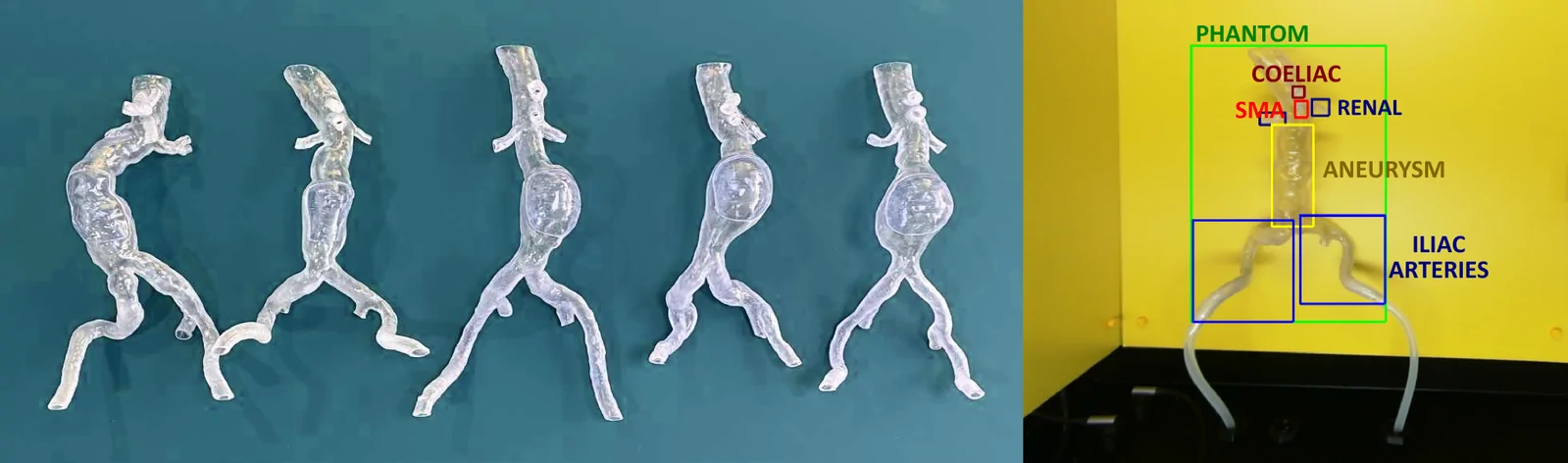
Left: 3D printed transparent aortic phantoms. Right: Phantom inside the simulator with bounding box annotations for the entire phantom, iliac arteries, renal arteries, aneurysm, superior mesencentric artery (SMA) and coeliac artery

We have 3D printed five transparent aortic phantoms, all of which exhibit abdominal aortic aneurysms of different sizes (see Fig. 3). The phantoms are based on five preoperative angio CTs of different patients (4 M, 1F), which were manually segmented by clinicians using Materialise Mimics Innovation Suite. On the ventral side of the phantoms, a removable window was placed at the position of the aneurysm in order to provide access to materials, making it easier to remove stent grafts after unfolding. The final 3D models were printed at 1:1 scale with a Polyjet printer (Stratasys Objet 350 Connex 3) from rigid, transparent polymer and were processed in an ultrasound bath. Prints resulting from this process were also subjected to a quality assessment routine in a high-resolution CT-scanner, finding the printing accuracy to be 70 μm compared to the original 3D volume.

### Scene object understanding

As shown in Fig. 1, the software side of PATRONUS is mainly concerned with gathering and fusing information from the simulator scene and the digital twin (CT) of the patient. Essentially, a homogeneous linear transform $\mathcal {T}_{camera,twin}$ is estimated that maps triangulated points inside the stereo camera scene to corresponding points in CT coordinates, using the aortic phantom as an anchor.

This mapping is established by reconstructing a 3D point cloud, which is then refined and registered to the original CT segmentation. From the stereo camera images, we first infer the relative depth using a disparity map generated by FoundationStereo [12], utilizing the stereo camera calibration we computed previously. The resulting point cloud $\mathcal {P}_{phantom}$ is filtered by separating the phantom from its background, which is achieved by masking the depth map with a *segmentation mask*. We retrieve this segmentation mask by prompting a foundation model (SAMv2 [17]) with the phantom bounding box.

The phantom bounding box is predicted by a custom trained YOLOv11 model. In particular, we train and compare different pre-trained versions and variants of YOLO [18] on a custom detection dataset $\mathcal {D}_{scene}$, finding that YOLOv11 yields the best performance. $\mathcal {D}_{scene}$ comprises 64 annotated images for training (2 patients), 43 images for validation (1 patient) and 16 images held out as a test set (2 patients). The models are trained with default settings and, in addition, various augmentations (HSV color shift, rigid transforms, flipping, mosaic). Each image is annotated with bounding boxes of multiple classes, representing anatomical landmarks, which are shown in Fig. 3 on the right.

The resulting isolated point cloud is downsampled, normalized and centered. We then uniformly sample a point cloud $\mathcal {P}_{seg}$ of 4000 points using Poisson disk sampling from the corresponding solidified 3D aortic segmentation as a target for our registration. An initial global alignment between $\mathcal {P}_{phantom}$ (moving) $\mathcal {P}_{seg}$ (fixed) is then estimated by mean-centering the normalized pointclouds. This initial alignment is further refined by the Iterative Closest Point (ICP) algorithm. In particular, we evaluate point-to-point ICP, point-to-plane ICP and generalized ICP for their suitability. In the end, we obtain a transform $\mathcal {T}_{camera,twin}$ from camera coordinates to CT coordinates of the digital twin anatomy by chaining the transforms recorded during this registration process.

**Fig. 4:**
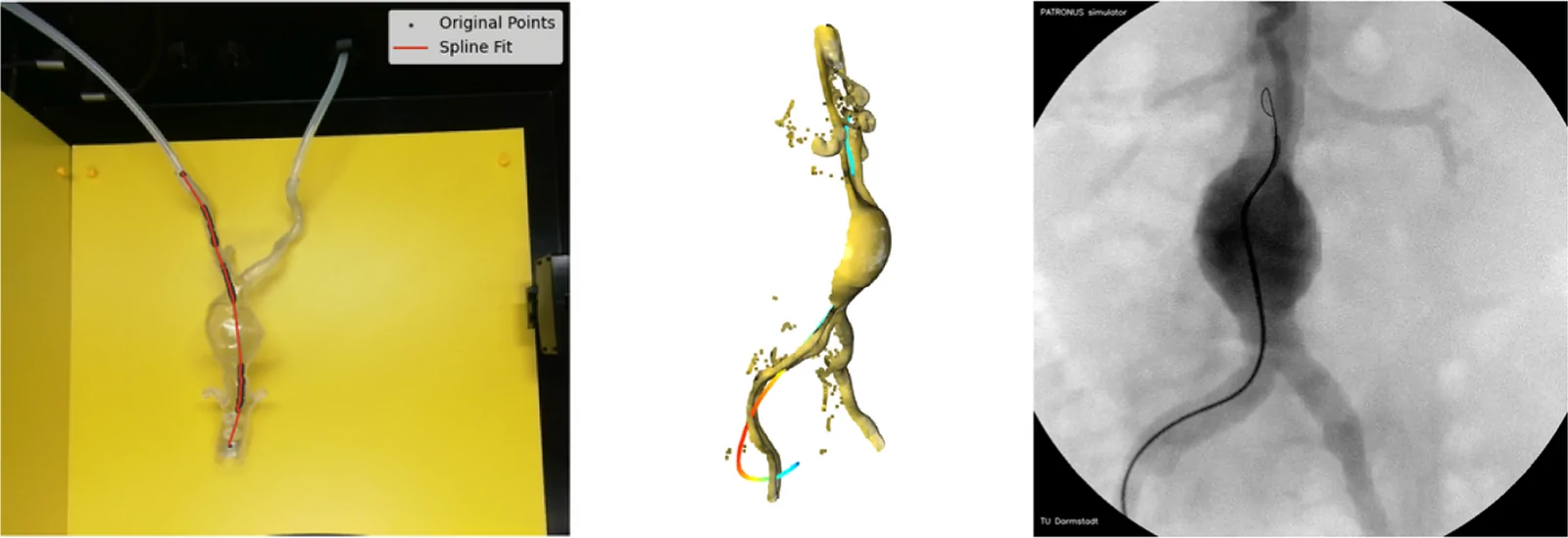
Left: Scene with reconstructed catheter spline. Center: Catheter spline in reconstruction space. Right: Rendered catheter on a pseudo X-ray

Since the inside of the simulator box is made of bright yellow panels and EVAR materials are typically dark, they are rather easy to distinguish on the camera images. Therefore, we can determine pixels that belong to the catheter by thresholding pixel values in HSV color space. For this purpose, we mask both images with the phantom segmentator (YOLO phantom detection prompting SAMv2). Since the resulting points are often scattered and sparse, we determine a smooth catheter trajectory shape by fitting a Smoothing B-Spline curve (s=0.2) through them (Fig. 4). We then match the resulting curve points from left and right images through nearest-neighbor correspondence (of curve points aligned on x-axis through median shifting) and triangulate them to obtain a set of 3D points representing the catheter trajectory.

### Pseudo x-ray generation

In the final stage of the PATRONUS pipeline, pseudo X-ray images are generated to be presented to the operator. Given the transform obtained previously through registration, we liken our X-ray detector to the camera in our blackbox simulator and therefore position the X-ray detector with a similar transform. Virtual contrast agent is injected into the phantom by directly changing the intensities of voxels within the vessel segmentation volume. The extracted catheter coordinates are sampled from the catheter B-Spline are transformed using $\mathcal {T}_{camera,twin}$ and the intensities inside the twin CT are manipulated at the resulting voxel positions.

Finally, a DRR of the in-silico EVAR scene is generated using DiffDRR [19], allowing for fast and appealing X-ray generation on the GPU. We use trilinear interpolation and patchification as additional performance improvements. We model a C-arm using a source-detector-distance of 102.0 cm, and generate images that are 400×400 pixels in size. The virtual gantry is oriented according to the transform estimated in Section 3.3.

## Results

**Fig. 5:**
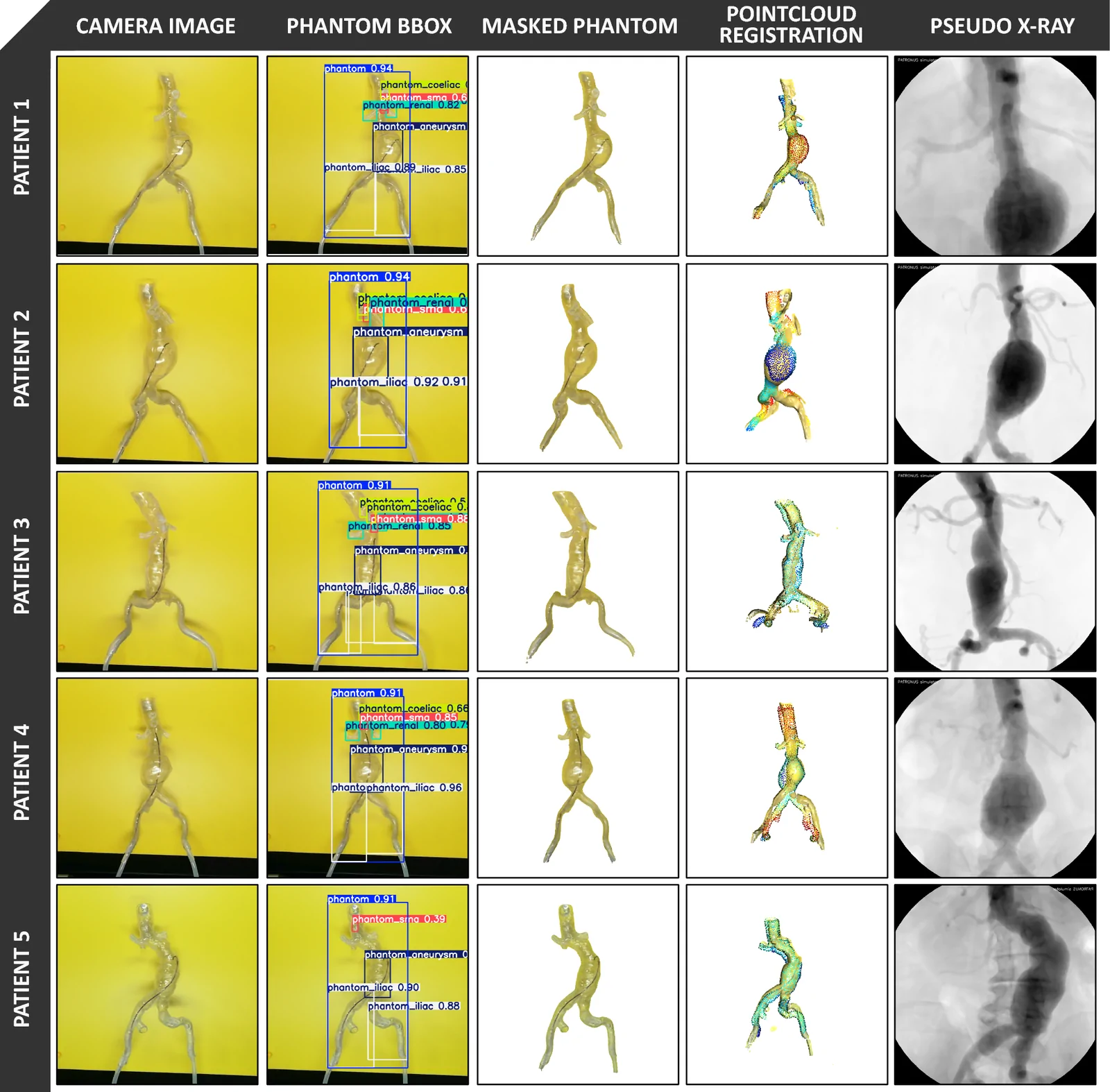
Qualitative results of the pseudo X-ray generation pipeline, illustrating the individual steps from the original camera image to the final pseudo X-ray. First column: Original camera image (left camera). Second column: Phantom bounding box (blue) and additional landmarks predicted by Yolo model. Third column: Phantom masked with SAM, prompted with predicted bounding box. Fourth column: Registration of 3D reconstruction and pre-operative vessel segmentation. Final column: Pseudo X-ray with simulated contrast agent

**Table 1:** Precision and recall per class for the YOLO detection models under comparison

Model/Class	Yolo11m		Yolo11s		Yolo11n		Yolo8m		Yolo8s		Yolo8n	
	P	R	P	R	P	R	P	R	P	R	P	R
Phantom	0.87	0.91	0.97	1.00	0.92	1.00	0.81	1.00	0.94	1.00	0.94	1.00
Iliac A	0.96	1.00	0.78	0.91	0.84	0.97	0.72	0.81	0.77	0.96	0.87	0.97
Renal A	0.91	0.97	0.82	0.91	0.92	0.87	0.72	0.88	0.84	0.91	0.83	0.88
Aneurysm	0.97	0.92	0.97	1.00	0.84	1.00	0.96	1.00	0.96	1.00	0.96	1.00
SMA	0.72	1.00	0.94	0.44	0.92	0.62	0.70	0.75	0.60	0.63	0.57	0.66
Coeiliac A	0.86	0.76	1.00	0.84	0.93	0.75	0.79	1.00	0.63	0.75	0.80	1.00

In the following, we outline the evaluation of the different pipeline stages used on the software side of our simulator setup, which we describe in Section 3. Concluding, we analyze the run time of our simulator pipeline.

The only trainable stage of our pipeline (Fig. 1) is the YOLO object detector, which is trained on different anatomical landmarks of the phantom scene. Different variants of YOLO are evaluated in Tab. 1. Furthermore, these landmarks can be used in the future to gather information about the position of materials, such as catheters, guidewires or stent grafts in relation to the patient’s anatomy, in order to align them with a pre-operative plan.

The segmentation stage is a SAMv2 model, prompted by the bounding box obtained in the prior step, reliably delineates the phantom for foreground separation. Reflections and transparency are not a challenge here, as can be seen in the qualitative results in Fig. 5.

**Fig. 6:**
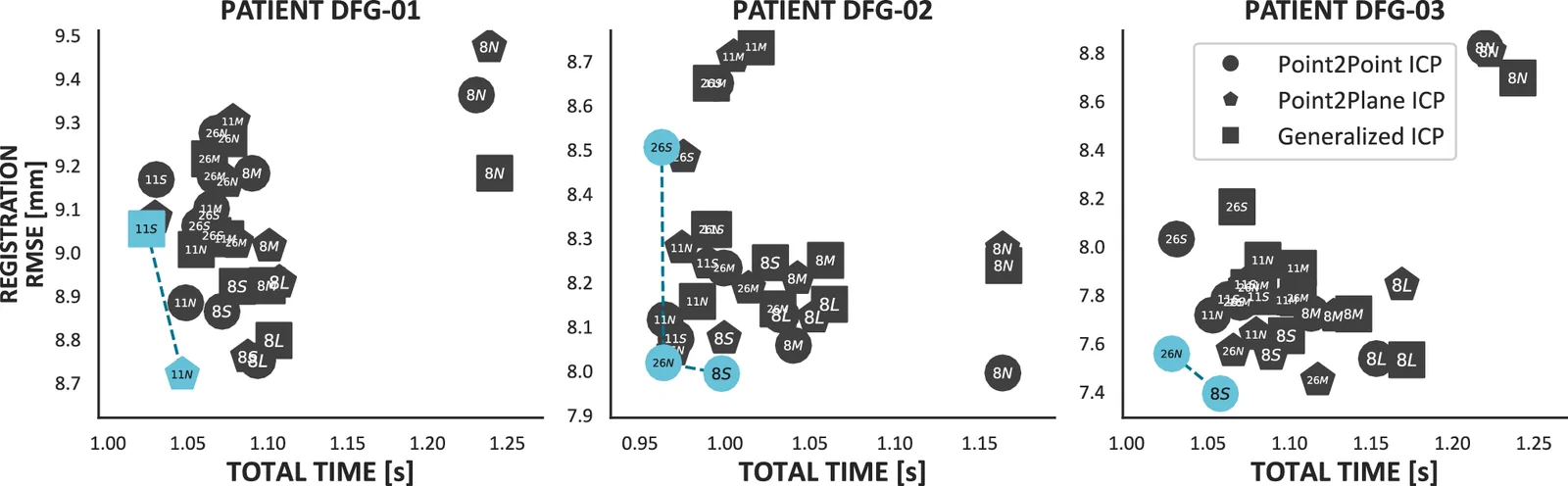
Comparison of different configurations of the registration pipeline for different phantoms, with respect to total wall clock execution time in seconds and final registration RMSE in millimeters. Different versions of YOLO (denoted inside scatter markers, zoom in for details) and variants of ICP (marker shape) are compared. The blue markers and curve show the Pareto optimal configurations and Pareto Front respectively

FoundationStereo works well for estimating the 3D positions of the phantoms, despite their transparency and their tendency to reflect light on the glossy surface. However, it is challenged by materials (catheters) inserted into the phantoms, which results in a structure on the point cloud surface that resembles a ravine. The cause for this issue lies in the bijective nature of depth estimation, as undercuts and objects behind transparent layers cannot be assigned two multiple depth values. However, in our experiments, this did not pose a challenge for computing the 3D-3D rigid alignment between point cloud $\mathcal {P}_{phantom}$ and CT. What is challenging for the rigid alignment are gaps in the point cloud, that are sometimes present if parts of the vasculature overlap and create undercuts, leaving a segmented point cloud. In future, we plan to fuse data of multiple stereo cameras (anterior-posterior, left-right projections) to enhance the 3D reconstruction of the scene, making it more robust to such artifacts.

On average, our entire pipeline takes an initial setup time of 14.56 s and takes 1.059 s to generate one pseudo X-ray image. Most of the generation time is taken by FoundationStereo, which takes 0.462 s on average to estimate a point cloud from a stereo image. Timings were evaluated on the host computer which is a desktop PC with an Intel Core i7-12700 CPU, 64 GB of RAM, and an NVidia GeForce RTX 3090 GPU.

There is a trade-off between runtime performance and accuracy depending on the registration pipeline’s configuration, which can be seen in Fig. 6. While this trade-off is not perfectly consistent among phantoms, there are some common configurations which balance this trade-off quite well. Point2Point ICP in combination with Yolo11N or Yolo8S for instance are typically close to the Pareto Front.

## Discussion & conclusion

In this work, we presented PATRONUS, a simulator setup for patient-specific surgical training for aortic aneurysm repair. We proposed a hardware setup based on 3D printed phantoms and stereo cameras, that enables patient-specific surgery on a black box phantom, and a data-driven software pipeline in which pseudo X-rays are generated to provide a realistic experience to the operator. This entirely eliminates the need for radiation during training, allowing for surgeons to practice in a safe environment. At the same time, anatomical features are displayed together with the vascular anatomy, which can be challenging (image artifacts) yet beneficial (anatomical cues for navigation).

Currently, our pipeline takes slightly more than one second to generate a pseudo X-ray on a consumer-grade GPU, which is already promising but demands further improvements in the future. This is a limitation of our work that we plan to address by investigating leaner stereo depth estimation models. Recently, Fast-FoundationStereo was published which makes use of self-distillation to increase runtime performance by up to factor 10 [20]. However, to the time of writing this paper, code and model weights of this model are not yet released but will be integrated into our comparison as soon as they are available.

On the hardware side, directions to improve are the simulator haptics and overall realism of surgical simulation in EVAR. In future, we plan to investigate perfusion of the phantom, and the potential for using flexible printing materials. One challenge is the effort of deploying additional hardware, keeping all electronics safe from leaking fluids. Flexible materials further pose the additional challenge that it is hardly feasible to 3D print flexible phantoms which are transparent enough for catheter tracking.

In perspective, these improvements to realism and performance will lead to a first set of user studies, targeting the evaluation of positioning accuracy and overall perceived utility of the simulator setup. In a second stage, capabilities for skill and error analysis will be integrated into the PATRONUS framework, enabled by the data collected within the simulated surgical scene. As catheters are tracked inside the simulator and are matched with a digital twin representation, it is possible to gather precise information about the tool locations during the simulated intervention. With future developments, the PATRONUS platform will allow to analyze the surgeon’s performance and potential risks during training, which is the main focus of this research endeavor. At this current stage of our research, we provide the hardware and software foundation for radiation-free and patient-specific training of EVAR, showing that this new simulation paradigm is generally feasible.
